# Long Non-Coding RNAs in Psoriasis: A Comprehensive Review of Expression Profiles, Mechanistic Insights, Genetic Associations, and Their Clinical Implications

**DOI:** 10.3390/ncrna11050069

**Published:** 2025-09-19

**Authors:** Judit Danis, Márta Széll

**Affiliations:** 1 Department of Immunology, Albert Szent-Györgyi Medical School, University of Szeged, H-6720 Szeged, Hungary; 2 HUN-REN-SZTE Dermatological Research Group, University of Szeged, H-6720 Szeged, Hungary; 3 Department of Medical Genetics, Albert Szent-Györgyi Medical School, University of Szeged, H-6720 Szeged, Hungary; szell.marta@med.u-szeged.hu; 4 HUN-REN-SZTE Functional Clinical Genetics Research Group, University of Szeged, H-6720 Szeged, Hungary

**Keywords:** long non-coding RNA, lncRNA, psoriasis, keratinocyte, epigenetic regulation, ceRNA, miRNA sponge, genetic susceptibility, clinical implications of lncRNAs

## Abstract

Psoriasis is a chronic inflammatory skin disorder affecting approximately 2% of the global population, characterized by abnormal keratinocyte proliferation and dysregulated immune responses. This review examines the emerging role of long non-coding RNAs (lncRNAs) in psoriasis pathogenesis, highlighting their significance as regulatory molecules in disease initiation, progression, and chronicity. LncRNAs demonstrate distinct expression patterns in psoriatic lesions, with upregulated transcripts such as MALAT1, XIST, MIR31HG, and HOTAIR promoting keratinocyte hyperproliferation, inhibiting apoptosis, and amplifying inflammatory cascades through mechanisms including microRNA sponging and transcription factor modulation. These molecules primarily target key signaling pathways including NF-κB, STAT3, and PI3K/AKT. Conversely, downregulated lncRNAs like NEAT1, MEG3, and PRINS normally function as tumor suppressor molecules that maintain epidermal homeostasis through pro-apoptotic and anti-inflammatory mechanisms. Their reduced expression contributes to the pathological hyperproliferative phenotype characteristic of psoriatic skin. Importantly, genetic variants within lncRNA loci have been identified as significant contributors to psoriasis susceptibility and treatment responses across different populations. Single- nucleotide polymorphisms in genes such as TRAF3IP2-AS1, HOTAIR, and CDKN2B-AS1 demonstrate population-specific associations with disease risk and therapeutic outcomes, suggesting their potential utility as pharmacogenomic markers. The complex regulatory networks involving lncRNAs provide new insights into psoriasis pathogenesis and offer promising avenues for personalized treatment strategies. Integration of lncRNA profiling into clinical practice may enhance our understanding of disease heterogeneity and improve therapeutic outcomes for psoriatic patients.

## 1. Introduction

Psoriasis is a complex, chronic inflammatory skin disorder that affects approximately 2% of the global population, representing a substantial public health burden [1,2]. The most common clinical form is plaque psoriasis, which is characterized by well-demarcated, erythematous plaques covered by silvery scales. These lesions result from abnormal proliferation and differentiation of basal keratinocytes in conjunction with a dysregulated immune response involving both innate and adaptive immune cells [3,4,5]. Importantly, psoriasis is not confined to visibly affected areas. The skin surrounding psoriatic plaques—referred to as non-lesional skin—appears clinically normal but harbors molecular and cellular changes consistent with a pre-psoriatic state [6,7,8,9,10,11,12,13,14]. These alterations include heightened sensitivity to stress signals [11,13,15] and increased expression of pro-inflammatory cytokines such as interleukin-1 (IL-1), tumor necrosis factor-alpha (TNF-α), and interferon-gamma (IFN-γ), even in the absence of visible inflammation [16]. Additionally, non-lesional skin contains elevated numbers of T-helper and suppressor cells, CD11b+ cells, and some subsets of dendritic cells, pointing to a primed immune environment [4,17,18,19]. The chronic inflammatory state of psoriasis is maintained by reciprocal interactions between keratinocytes and immune cells. Infiltrating leukocytes, especially IL-17-producing T-helper cells, release pro-inflammatory mediators that exacerbate keratinocyte hyperproliferation and disturb epidermal homeostasis [3,4]. These processes generate self-reinforcing loops of inflammation and epidermal remodeling that not only drive disease progression but may also explain the persistence and recurrence of lesions in the same anatomical regions [16,20]. Beyond the skin, psoriasis is associated with systemic comorbidities, including metabolic syndrome, cardiovascular disease, and psychological disorders, particularly in adolescents and young adults [2,21].

In the last two decades, high-throughput gene expression studies have fundamentally shaped our understanding of psoriasis pathogenesis [22,23,24]. These investigations have also highlighted the critical roles of non-coding RNAs (ncRNAs) as important molecular players in disease development and progression [25,26,27]. NcRNAs represent diverse molecules, which do not translate into proteins, and were long considered as non-functional transcriptional noise. However, the past two decades revealed their crucial roles in regulating cellular processes. Our understanding of ncRNAs began in the early 2000s, with the unexpected discovery of numerous small RNA species, such as small interfering RNAs (siRNAs), microRNAs (miRNAs), and small PIWI-interacting RNAs (piRNAs), which regulate gene expression at transcriptional, post-transcriptional, and translational levels [28].

Among ncRNAs, long non-coding RNAs (lncRNAs) emerged as particularly important regulators. These longer transcripts exhibit diverse sequence features, structures, and functions, with the current consensus defining them as transcripts longer than 500 nucleotides in length, primarily transcribed by RNA polymerase II [28]. LncRNAs interact with DNA, RNA, and protein complexes to influence transcriptional regulation, chromatin remodeling, and post-transcriptional modifications including splicing and mRNA stability. In the early 2000s, PRINS non-coding RNA was one of the first ncRNAs identified in psoriasis as a lncRNA conferring susceptibility to the disease [11]. Since then, our knowledge on lncRNAs has widely broadened. While, initially, research focused on expression profiles of lncRNAs in various diseases, in the last decade, their specific, disease-related functions were also highlighted. In psoriasis, both cellular lncRNAs [29] and circulating lncRNAs [30,31] are deregulated. Both keratinocytes and immune cells show deregulated lncRNA expression patterns; however, keratinocytes show the highest number of differentially expressed lncRNAs [29].

An expanding body of literature highlights the role of lncRNAs in shaping the transcriptomic and epigenetic landscape of psoriatic skin, contributing to both lesion formation and disease persistence [27,32,33,34,35]. These findings indicate that lncRNAs serve not only as markers of disease activity but may also function as potential therapeutic targets.

Here, we provide a summary on the current knowledge on the involvement of long non-coding RNAs in psoriasis, exploring their roles in disease initiation, progression, chronicity, and genetic predisposition, to provide a comprehensive perspective on how lncRNAs contribute to the multifactorial nature of this immune-mediated skin disease.

## 2. Literature Search Strategy

The relevant literature was identified using scite.ai with the search prompt “*lncRNA AND psoriasis*” up to June 2025. Each article was assessed for relevance, and only studies providing original data from human psoriatic tissues, keratinocytes, or blood samples were included. From these primary sources, we compiled a comprehensive list of all lncRNAs reported to date in psoriasis. To ensure completeness, we subsequently conducted targeted PubMed searches combining the name of each identified lncRNA with the keyword “*psoriasis*”, which allowed us to capture any additional relevant publications. This strategy ensured inclusion of all experimentally supported lncRNAs in psoriasis while excluding speculative reports without direct patient-derived evidence.

## 3. Dysregulated Expression of Long Non-Coding RNAs in Psoriasis

### 3.1. LncRNAs Upregulated in Psoriasis

**Figure 1:**
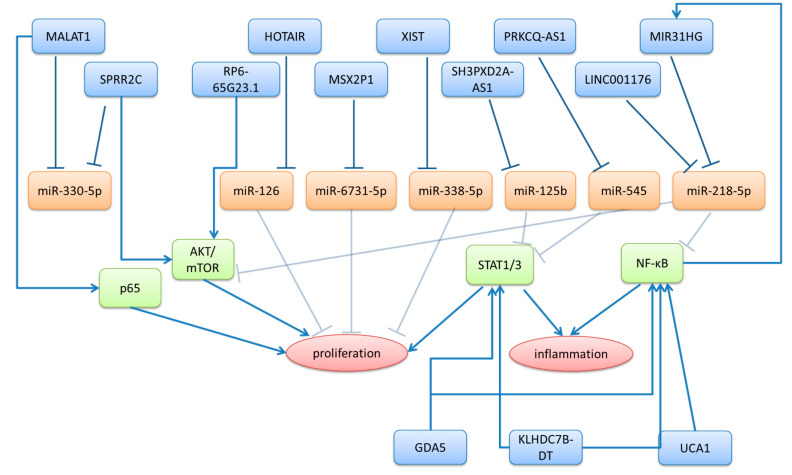
The regulatory network of lncRNAs upregulated in psoriasis. Upregulated lncRNAs in psoriasis (blue) mostly inhibit miRNA (orange) functions by acting as ceRNAs (blue, bar-headed arrows). This sponging of miRNAs eliminates their negative regulatory functions (gray bar-headed arrows), thus contributing to the upregulation of several psoriasis-related pathways, such as AKT/mTOR, JAK-STAT, and NF-κB (green), while some lncRNAs also directly interact (blue arrows) and activate these pathways. These upregulated pathways amplify the proliferation and inflammation of skin cells, characteristic for psoriatic plaques. (Blue: upregulates lncRNAs in psoriasis; orange: lncRNA-regulated miRNAs; green: transcription factors and pathways regulated by miRNAs and lncRNAs; red: affected cellular processes; blue bar-headed arrows: inhibition; gray bar-headed arrows: inhibitory activity, which is eliminated by upstream regulation by ceRNAs; blue arrows: activation).

Long non-coding RNAs with increased expression in psoriatic lesions typically function as oncogenes, promoting keratinocyte hyperproliferation, inhibiting apoptosis, and amplifying inflammatory signaling cascades (Table 1 and Figure 1). These upregulated lncRNAs often act through complex regulatory networks involving diverse mechanisms. One common mode of action is competing endogenous RNAs (ceRNAs), also referred to as miRNA sponging. In this mechanism, lncRNAs bind specifically to miRNAs at their mRNA target sites, competing with mRNAs for miRNA binding. This sequestration can lead to miRNA degradation and, consequently, the upregulation of target mRNAs [36]. Another potent mechanism of lncRNAs is a direct protein interaction to modulate their functions. This is particularly relevant in the case of key transcription factors such as NF-κB and STAT3 [37]. The elevated expression of these lncRNAs contributes to the characteristic features of psoriatic plaques, including epidermal thickening, altered differentiation programs, and sustained inflammatory responses. Interestingly, some upregulated lncRNAs show tissue-specific expression patterns, with differential regulation between skin lesions and systemic circulation [38,39,40], suggesting compartmentalized roles in local versus systemic disease manifestations.

Several long non-coding RNAs demonstrate significant upregulation in psoriatic lesions and contribute to disease pathogenesis through diverse molecular mechanisms. MALAT1 (Metastasis Associated Lung Adenocarcinoma Transcript 1) shows increased expression in both lesional and non-lesional skin as well as the serum of psoriatic patients [41]. Mechanistically, MALAT1 is upregulated by IL-17, one of the master cytokines in psoriasis. MALAT1 can directly bind to p65 transcription factor, facilitating the proliferation of keratinocytes [42]. Moreover, MALAT1 acts as a ceRNA for miR-330-5p, a regulator of S100A7 expression [43]. S100A7 belongs to the S100 gene family, and is an antimicrobial protein that exerts chemotactic and pro-inflammatory action and is highly expressed in psoriatic lesions [44].

Similarly, XIST (X-Inactive-Specific Transcript) is elevated in serum of psoriatic patients and demonstrates proproliferative effects in keratinocytes [45]. Overexpression of XIST in HaCaT cells significantly enhances cell proliferation while also promoting the release of inflammatory cytokines [45,46]. Moreover, XIST also acts as a ceRNA for several miRNAs, and is a part of an lncRNA–ceRNA regulatory network [47], suggesting its role in perpetuating the inflammatory cascade characteristic of psoriasis.

MIR31HG represents another significantly upregulated lncRNA in lesional psoriatic skin, with its expression being induced by NF-κB signaling. Functional studies reveal that siRNA-mediated MIR31HG depletion induces cell cycle arrest in the G2/M phase, highlighting its importance in the control of keratinocyte proliferation [48].

Additionally, LINC01176 shows significant elevation in psoriatic samples and also acts as a ceRNA for miR-218-5p. Mir-218-5p targets multiple signaling pathways [49,50] and IL-36γ; thus, when LINC01176 binds to mir-218-5p, the expression of IL-36γ increases. The downstream effects of a higher LINC01176 expression on IL-36γ expression promote the hyperproliferative phenotype of psoriatic keratinocytes through proliferation, invasion, and inhibition of apoptosis [51]. Moreover, the shRNA-mediated in vivo knockdown of LINC01176 efficiently reduced the psoriatic symptoms in the imiquimod (IMQ) induced mouse model [51].

HOTAIR (HOX Transcript Antisense RNA) also exhibits elevated levels in serum samples of psoriatic patients [52] and plays a crucial role in keratinocyte apoptosis regulation. HOTAIR binds to miR-126 and acts as a ceRNA. Overexpression of HOTAIR induces apoptosis in IL-22-stimulated HaCaT keratinocytes, an effect that can be reversed by miR-126 [53]. Interestingly, miR-126 is inversely expressed in psoriatic skin and serum, with high expression in psoriatic keratinocytes and lower expression in serum samples of the patients [54,55,56], suggesting a complex regulatory network involving cytokine signaling and ceRNA-mediated gene regulation [53].

### 3.2. Downregulated lncRNAs in Psoriasis

**Figure 2:**
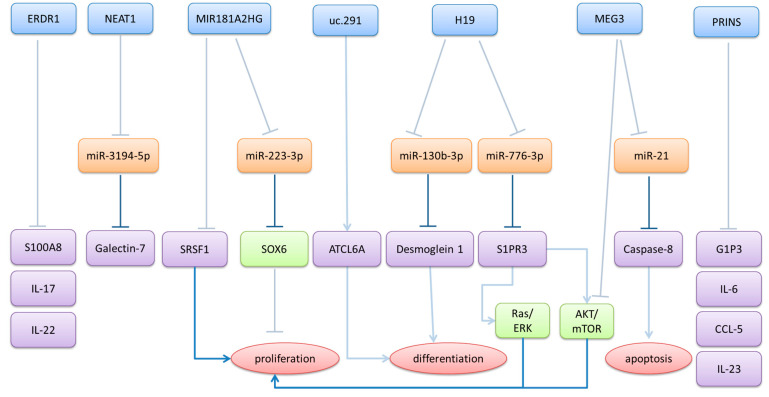
The regulatory network of lncRNAs downregulated in psoriasis. Downregulated lncRNAs in psoriasis (blue) cannot exert their ceRNA-mediated inhibitory effects (gray, bar-headed arrows) on miRNAs (orange). This allows for miRNAs to inhibit the expression of their target genes (purple), subsequently activating signaling pathways, such as SOX6, Ras/ERK, and AKT/mTOR, leading to defects in differentiation, apoptosis, and an increase in proliferation. Some lncRNAs (e.g., ERDR1, PRINS) also directly inhibit their target genes (grey, bar headed arrows). Blue: downregulated lncRNAs in psoriasis; orange: lncRNA-regulated miRNAs; green: transcription factors and pathways regulated by miRNAs and lncRNAs; purple: genes regulated by miRNAs and lncRNAs; red: affected cellular processes; blue bar-headed arrows: inhibition.; gray bar-headed arrows: inhibitory activity eliminated by the downregulation of lncRNAs; blue arrows: activation).

Downregulated long non-coding RNAs in psoriatic lesions (Table 2 and Figure 2) generally function as tumor suppressors, normally serving to maintain epidermal homeostasis through the promotion of apoptosis, regulation of cell cycle progression, and suppression of inflammatory responses [57]. The loss of or reduction in these protective lncRNAs contributes to the pathological features of psoriasis by removing critical regulatory checkpoints that normally prevent excessive keratinocyte proliferation and inflammatory cytokine production. Many of these downregulated lncRNAs demonstrate complex tissue-specific expression patterns, with some showing paradoxical upregulation in circulating blood cells while being suppressed in skin lesions, suggesting distinct roles in local tissue regulation versus systemic immune responses. The functional restoration of these suppressed lncRNAs represents a potential therapeutic strategy for psoriasis treatment.

NEAT1 (Nuclear Enriched Abundant Transcript 1) demonstrates decreased expression specifically in skin samples of psoriatic patients [20,40], despite being increased in blood samples [38,39]. This tissue-specific regulation suggests complex regulatory mechanisms, which might be part of an lncRNA-miRNA regulatory network in psoriasis [47]. NEAT-1 targets several miRNAs, including miR-3194-5p, which regulates galectin-7 expression in the skin microenvironment [40].

MEG3 (Maternally Expressed Gene 3) is consistently downregulated in psoriatic skin samples and regulates miR-21 expression [58,59]. MEG3 and miR-21 are suggested as potential biomarkers for psoriasis, while MEG3 also show distinct expression-patterns in therapy-resistant patient samples [59,60]. The targeting of miR-21 by MEG3 exerts apoptotic effects [58]; however, MEG3 also suppresses inflammation, promotes autophagy, and inhibits the PI3K/AKT/mTOR signaling pathway [61]. The data altogether suggest that its downregulation contributes to the anti-apoptotic and hyperproliferative state of psoriatic keratinocytes [61].

One of the first identified lncRNAs in psoriasis was PRINS (Psoriasis Susceptibility-Related RNA Gene Induced by Stress) [11]. PRINS exhibits tissue-specific regulation in psoriatic skin samples with high expression in non-lesional epidermis and lower expression in lesional epidermis samples when compared to the non-lesional paired samples [11,62]. Meanwhile, expression in serum samples is downregulated [30]. Association of high PRINS expression in the buccal epithelium of psoriatic children with disease severity was also recently suggested [63]. PRINS was shown to affect cellular stress responses through regulating the functions of nucleophosmin (NPM1) and the expression of the anti-apoptotic protein G1P3 in keratinocytes [64]. Moreover, PRINS also exerts anti-inflammatory effects, by targeting the mRNAs of IL-6 and CCL-5 [65,66] and regulating IL-23 expression in keratinocytes [67], suggesting that its upregulation in psoriatic non-lesional epidermis contributes to the maintenance of non-lesional skin phenotype.

**Table 1:** LncRNAs with upregulated expression in psoriasis and their disease-related functions.

lncRNA	Expression in Psoriasis	Psoriasis-Related Functions of lncRNA	Reference
AC020916.1	upregulated in lesional skin	expression correlates with expression of cell proliferation/epidermal differentiation genes	[29]
AGXT2L1-2:2	upregulated in psoriatic keratinocytes	promotes proliferation of keratinocytes and inhibits apoptosis	[68,69]
CYDAER	upregulated in psoriatic keratinocytes	upregulated by IL-17A and during early differentiation downregulated in late differentiation stages of keratinocytes knockdown of CYDAER promotes terminal differentiation	[35]
GAS5	upregulated in psoriatic serum		[31]
GDA-5	upregulated in lesional skin	regulates FOXM1 expression promotes proliferation and inflammation in keratinocytes via STAT3 and NF-κB signaling	[70]
HOTAIR	upregulated in psoriatic serum	regulates apoptosis by acting as a ceRNA for miR-126	[52,53]
KLHDC7B-DT	upregulated in lesional skin	promotes proliferation and inflammatory cytokine secretion (IL-6, IL-8) through STAT3 and NF-κB signaling	[71]
LINC00958	upregulated in psoriatic keratinocytes	promotes proliferation of keratinocytes	[27]
LINC01176	upregulated in lesional skin	promotes proliferation of keratinocytes acts as a ceRNA for miR-218-5p knockdown alleviated psoriasiform symptoms in IMQ-induced mouse model of psoriasis	[51]
LINC01215	upregulated in lesional skin	expression correlates with response to biological therapy	[72,73]
LINC1206	upregulated in lesional skin		[72]
MALAT1	upregulated in lesional and non-lesional skin and psoriatic serum	promotes proliferation of keratinocytes by binding to p65 regulates S100A7 expression by acting as a ceRNA for miR-330-5p	[41,42,43]
MIR31HG	upregulated in lesional skin	promotes proliferation of keratinocytes	[48]
MSX2P1	upregulated in lesional skin	promotes proliferation of keratinocytes by acting as a ceRNA for miR-6731-5p	[74]
PRKCQ-AS1	upregulated in lesional skin	regulates JAK-STAT1 pathway by acting as a ceRNA for miR-545 exosomal PRKCQ-AS1 from keratinocytes promotes Th17 differentiation of CD4+ T-cells	[75,76]
RP6-65G23.1	upregulated in lesional skin	promotes proliferation and inhibits apoptosis via p-ERK1/2/p-AKT signaling pathway	[25,77]
SH3PXD2A-AS1	upregulated in lesional skin	promotes proliferation and suppresses apoptosis in keratinocytes regulates STAT3 expression by acting as ceRNA for miR-125b	[72,75,78]
SPRR2C	upregulated in lesional skin and psoriatic keratinocytes	promotes proliferation of keratinocyte, by increasing STAT1 and S100A7 expression through acting as a ceRNA for miR-330-5p and by regulating the PI3K/AKT/mTOR signaling pathways	[79,80]
SPRR2G-2	upregulated in lesional skin and psoriatic keratinocytes	promotes proliferation and inflammation by activating the STAT3 pathway	[81]
UCA1	upregulated in lesional skin	positively regulates keratinocyte-driven inflammation via the NF-κB and HIF-1α pathways	[82]
XIST	upregulated in psoriatic serum	regulates proliferation and inflammation of keratinocytes, by acting as a ceRNA for miR-338-5p	[45,46]

**Table 2:** LncRNAs with downregulated expression in psoriasis and their disease-related functions.

lncRNA	Expression in Psoriasis	Psoriasis Related Functions of lncRNA	Reference
ERDR1	downregulated in lesional skin	in IMQ-induced model, recombinant ERDR1 decreases psoriasis-like symptoms and expression of IL-17, IL-22, and S100A8	[83,84]
H19	downregulated in lesional skin	regulates proliferation and inflammation through the H19/miR-766-3p/S1PR3 axis regulates differentiation by acting as a ceRNA for miR-130b-3p	[85,86]
LINC00941	downregulated in lesional skin		[87]
LOC100130476	downregulated in lesional skin		[25]
LOC285194	downregulated in lesional skin		[88,89]
MEG3	downregulated in lesional skin MEG3 and miR-21 are possible diagnostic markers for psoriasis	promotes apoptosis by acting as a ceRNA for miR-21 suppresses inflammation and promotes autophagy through the PI3K/AKT/mTOR pathways	[58,59,60,61]
MIR181A2HG	downregulated in lesional skin	regulates proliferation of keratinocytes, by acting as a ceRNA for miR-223-3p and by binding to SRSF1	[90,91]
NEAT1	increased NEAT-1 expression in psoriatic blood samples downregulated in lesional skin downregulated in resolved epidermis compared to never lesional epidermis	regulates expression of galectin-7 by acting as a ceRNA for miR-3194-5p	[20,38,39,40]
PRINS	upregulated in non-lesional skin and in psoriatic keratinocytes downregulated in psoriatic serum, along with its interactors G1P3 and NPM-1 high PRINS expression in the buccal epithelium with low expression in psoriatic keratinocytes lead to severe psoriasis with prolonged exacerbation and unfavorable prognosis	regulates G1P3 expression in keratinocytes binds to NPM-1 and regulates the UVB-induced nucleolar shuttling of NPM-l downregulates IL-6 and CCL-5 expression, by binding to the respective mRNAs and downregulates IL-23a in keratinocytes	[11,30,62,63,64,65,66,67]
uc.291	downregulated in lesional skin	regulates differentiation by interacting with ATCL6A promotes proliferation of keratinocytes	[92,93]
7SL-RNA	downregulated in lesional skin		[94]

## 4. Genetic Associations and Clinical Implications of lncRNAs in Psoriasis

Psoriasis has a strong genetic component, which is reflected in twin studies. Studies have shown that monozygotic twins have a higher concordance rate for psoriasis (65–72%) than dizygotic twins (15–30%). While genetic factors play a significant role in the development of psoriasis, the rest of the variation can be explained by environmental factors [95]. Originally, variants in protein coding regions were shown to be associated with psoriasis susceptibility. Human leukocyte antigen (HLA) genes, notably HLA-C*06:02, represent the most potent genetic risk factors for psoriasis development and therapy response [96]. In recent decades, large-scale genome-wide association studies (GWASs) have led to an increase in the number of genetic loci associated with psoriasis susceptibility [97].

Lately, genetic variants within long non-coding RNA loci have also emerged as significant contributors to psoriasis susceptibility, treatment response, and disease severity, highlighting the importance of these regulatory elements in inherited disease risk (Table 3). Single-nucleotide polymorphisms (SNPs) in lncRNA genes can alter their expression levels, structural stability, or interaction capabilities with other regulatory molecules, thereby modifying disease phenotypes [98]. Interestingly, most identified variants lie within lncRNAs already showing altered expression patterns in psoriasis, highlighting the complex regulatory role of lncRNAs in the disease. Population-specific genetic associations across different ethnic groups suggest that lncRNA-mediated disease mechanisms may vary between populations and could influence personalized treatment approaches. Additionally, some lncRNA variants specifically associate with therapeutic responses, indicating their potential utility as pharmacogenomic markers for optimizing treatment selection in psoriatic patients.

HOTAIR is not only upregulated in psoriasis but also harbors multiple disease-associated variants. The variant rs12826786 showed association with psoriasis risk in both Iranian [99] and Chinese Han [100] population, while rs4759314 demonstrates additional risk association only in the Chinese Han population [100]. These genetic associations complement the functional data showing HOTAIR upregulation in psoriatic serum [52] and its role as a ceRNA for miR-126 [53].

MALAT1 contains the rs619586 polymorphism, which has been identified as a risk locus for psoriasis in the Iranian population [101], further supporting the functional relevance of this lncRNA in disease pathogenesis.

*TRAF3IP2* encodes Act1, a regulator of IL-17 signaling. In response to IL-17 stimulation, Act1 is recruited to the IL-17R complex and subsequently activates the NF-κB, MAPK, and C/EBP signaling pathways; thus, it is an essential step in the activation of IL-17A-mediated signaling [102]. A psoriasis-susceptible variant rs13210247 is located in the exon of a lncRNA TRAF3IP2-AS1, an anti-sense lncRNA of *TRAF3IP2* [103,104,105,106]. TRAF3IP2-AS1 negatively regulates Act1 expression and subsequently IL-17 signaling. The variant rs13210247 is a gain-of-function mutant, with enhanced ability to inhibit IL-17 signaling compared to the wild-type variant [102].

The CDKN2B-AS1 (ANRIL) locus demonstrates multiple psoriasis-associated variants, most of which are associated with a higher risk for psoriasis, while the rs4977574 appears to confer protective effects against the disease [107,108]. Variants of ANRIL are also linked to response to apremilast therapy [109]. Similarly, LINC00941, an lncRNA showing decreased expression in psoriatic skin samples [87], contains rs12297445, a variant associated with treatment response to apremilast [109]. These observations demonstrate the important clinical implications of lncRNA-associated genetic markers. These results can be utilized in a personal medicine approach; however, with additional findings on other lncRNAs, their genotyping could be included in future therapeutic approaches.

Interestingly, most genetic association studies of lncRNAs in psoriasis were performed in Chinese Han and Iranian populations. While these cohorts provide valuable insights, their findings may not necessarily be generalizable to global populations, given the ethnic heterogeneity observed in psoriasis genetics. Multi-ethnic GWASs have demonstrated that certain susceptibility loci have strong associations in one ethnic group but are rare in others [110]. Therefore, replication studies across diverse populations are crucial to determine whether the lncRNA-associated variants observed thus far are globally relevant or represent ethnic-specific genetic effects.

**Table 3:** Known genetic variants of lncRNAs associated with psoriasis susceptibility or treatment response.

lncRNA	Associated Variant	Type of Association	Publications
ANRIL	rs1063192	associated with response to apermilast therapy	[109]
	rs1333048	more prevalent in psoriatic patients	[108]
	rs10120688	associated with response to apermilast therapy	[109]
	rs10757178	more prevalent and increased risk in psoriatic patients	[108]
	rs2518723	more prevalent in psoriatic patients	[107]
	rs3217992	more prevalent in psoriatic patients	[107]
	rs4977574	protective against psoriasis	[108]
HOTAIR	rs12826786	increased risk of psoriasis	[99]
	rs4759314	increased risk of psoriasis	[99,100]
LINC00941	rs12297445	associated with response to apermilast therapy	[109]
MALAT1	rs619586	increased risk of psoriasis	[101]
TARF3IP2-AS1	rs13210247	increased risk of psoriasis	[102,103,104,105,106]

## 5. Therapeutic Targeting of lncRNAs in Psoriasis

To our knowledge, there are currently no human clinical trials that are directly targeting lncRNAs in psoriasis. However, preclinical in vivo evidence supports that modulating specific lncRNAs can ameliorate psoriasiform disease in mouse models. Particularly, the lentiviral delivery of the mouse homolog of TRAF3IP2-AS1 (*E130307A14-Rik*), a regulator of IL-17 signaling, yielded therapeutic effects in an IMQ-induced mouse model of psoriasis, by reducing epidermis thickness, immune cell infiltration, and pro-inflammatory cytokine expression [102]. Similarly, the delivery of recombinant ERDR1 was also able to significantly reduce psoriasiform symptoms and the expression of IL-17, IL-22, and CCL20 in IMQ-induced mouse models of psoriasis [84].

The shRNA-mediated knockdown of LINC01176 in the IMQ-induced mouse model of psoriasis also had therapeutic effects by reducing plaque formation and the expression of inflammatory cytokines, such as IL-17, IL-23, TNF-α, and IL-36γ [51].

These results show the potential treatment capability of targeting lncRNAs in psoriasis. However, there are still difficulties in skin-directed lncRNA therapies pertaining to the efficient and durable cutaneous delivery of recombinant lncRNAs or antisense oligonucleotides for targeting relevant lncRNAs [111,112].

## 6. Conclusions

Long non-coding RNAs (lncRNAs) have emerged as critical regulators in the pathogenesis of psoriasis, influencing cellular functions, inflammatory responses, and immune cell interactions. Most studies focus on the role of lncRNAs in keratinocytes, since these cell types have the most deregulated lncRNAs in the disease. Highly expressed lncRNAs in psoriatic lesions usually promote disease-associated pathways, including inflammation and keratinocyte proliferation. These molecules exert their effects through acting as a ceRNA, modulation of transcription factors, and interaction with signaling cascades such as NF-κB, STAT3, and PI3K/AKT. Downregulated transcripts mainly have anti-inflammatory or pro-apoptotic and antiproliferative functions, and due to their low expression, they cannot exert their normal functions in psoriatic cells. Through these mechanisms, both upregulated and downregulated lncRNAs contribute to the chronic inflammatory and hyperproliferative phenotype characteristics of psoriatic skin. Furthermore, genetic variants within lncRNA loci have been linked to disease susceptibility and treatment responses, underscoring their relevance as biomarkers and potential therapeutic targets.

Therapeutic manipulation of lncRNAs remains at an early stage, but RNA-directed medicines are beginning to demonstrate translational potential in inflammatory diseases. For example, obefazimod (ABX464)**,** a small molecule that modulates the splicing of lncRNA 0599-205 to enhance miR-124 expression [113], has shown efficacy in late-phase clinical trials for ulcerative colitis, leading to suppression of IL-6 and IL-17A pathways [114,115]. Although not yet explored in psoriasis, such a proof of concept illustrates that targeting the lncRNA–microRNA axes is both feasible and clinically relevant, opening a new avenue for therapeutic innovation.

It is important to note that most available studies on lncRNAs in psoriasis are based on single-center, relatively small patient cohorts and remain exploratory. Variability in study design, sample source (e.g., blood, skin, keratinocytes), and analytic methods also limits direct comparability between findings. While we provide a comprehensive overview, the strength of evidence for individual lncRNAs varies, and for many lncRNAs, further validation in larger, independent cohorts will be necessary to establish their biological and clinical relevance. Overall, the growing body of evidence supports the integration of lncRNA profiling into psoriasis research and personalized treatment strategies, offering new avenues for understanding disease heterogeneity and improving clinical outcomes.

## Abbreviations

The following abbreviations are used in this manuscript:

AKT	Protein kinase B
AMPK	AMP-activated protein kinase
ANRIL	CDKN2B-AS1, antisense non-coding RNA in the INK4 locus
CCL-5	C-C motif chemokine ligand 5
CCL20	C-C motif chemokine ligand 20
C/EBP	CCAAT enhancer binding protein
ceRNA	Competing endogenous RNA
DC	Dendritic cell
ERDR1	Erythroid differentiation regulator 1
FOXM1	Forkhead box protein M1
G1P3	Interferon, alpha-inducible protein 6 (IFI6)
GAS5	Growth arrest-specific 5
HIF-1α	Hypoxia-inducible factor 1 alpha
HOTAIR	HOX transcript antisense RNA
IL-17	Interleukin-17
IL-22	Interleukin-22
IL-23	Interleukin-23
IL-36G	Interleukin-36 gamma
IL-6	Interleukin-6
IL-8	Interleukin-8
IMQ	Imiquimod
JAK	Janus kinase
LINC00958	Long intergenic non-protein coding RNA 958
LINC01176	Long intergenic non-protein coding RNA 1176
lncRNA	Long non-coding RNA
MALAT1	Metastasis associated lung adenocarcinoma transcript 1
MAPK	Mitogen-activated protein kinase
MEG3	Maternally expressed gene 3
miRNA	MicroRNA
mRNA	Messenger RNA
ncRNA	Non-coding RNA
NF-κB	Nuclear factor kappa-light-chain-enhancer of activated B cells
NPM-1	Nucleophosmin 1
p65 (RELA)	NF-κB subunit p65
p-ERK1/2	Phosphorylated extracellular signal-regulated kinase 1/2
PI3K	Phosphoinositide 3-kinase
PRINS	Psoriasis susceptibility-related RNA gene induced by stress
PRKCQ-AS1	Protein kinase C theta antisense RNA 1
RISC	RNA-induced silencing complex
S100A7	S100 calcium-binding protein A7 (psoriasin)
S1PR3	Sphingosine-1-phosphate receptor 3
shRNA	Short hairpin RNA
siRNA	Small interfering RNA
SNP	Single-nucleotide polymorphism
SOX6	SRY-box transcription factor 6
SPRR2C	Small proline-rich protein 2C
SRSF1	Serine/arginine-rich splicing factor 1
STAT1	Signal transducer and activator of transcription 1
STAT3	Signal transducer and activator of transcription 3
Th17	T-helper 17 cell
TNF-α	Tumor necrosis factor-alpha
TRAF3IP2	TNF receptor-associated factor 3
TRAF3IP2-AS1	TRAF3IP2 antisense RNA 1
UCA1	Urothelial carcinoma-associated 1
UVB	Ultraviolet B radiation
XIST	X-inactive-specific transcript
